# The impact of evacuation on the incidence of chronic kidney disease after the Great East Japan Earthquake: The Fukushima Health Management Survey

**DOI:** 10.1007/s10157-017-1395-8

**Published:** 2017-03-15

**Authors:** Yoshimitsu Hayashi, Masato Nagai, Tetsuya Ohira, Hiroaki Satoh, Akira Sakai, Akira Ohtsuru, Mitsuaki Hosoya, Yukihiko Kawasaki, Hitoshi Suzuki, Atsushi Takahashi, Yoshihiro Sugiura, Hiroaki Shishido, Hideto Takahashi, Seiji Yasumura, Junichiro James Kazama, Shigeatsu Hashimoto, Gen Kobashi, Kotaro Ozasa, Masafumi Abe

**Affiliations:** 1 Radiation Medical Science Center for the Fukushima Health Management Survey, Fukushima Medical University, Fukushima, Japan; 2 Department of Nephrology and Hypertension, Fukushima Medical University, 1 Hikarigaoka, Fukushima City, 960-1295 Japan; 3 Department of Epidemiology, Fukushima Medical University, Fukushima, Japan; 4 Department of Diabetology, Endocrinology, and Metabolism, Fukushima Medical University, Fukushima, Japan; 5 Department of Radiation Life Sciences, Fukushima Medical University, Fukushima, Japan; 6 Department of Radiation Health Management, Fukushima Medical University, Fukushima, Japan; 7 Department of Pediatrics, Fukushima Medical University, Fukushima, Japan; 8 Department of Cardiology, Fukushima Medical University, Fukushima, Japan; 9 Department of Gastroenterology, Fukushima Medical University, Fukushima, Japan; 10 Department of Neurology, Fukushima Medical University, Fukushima, Japan; 11 Department of Orthopedics, Fukushima Medical University, Fukushima, Japan; 12 Department of Public Health, Fukushima Medical University, Fukushima, Japan; 13 Department of Public Health, Dokkyo Medical University, Mibu, Japan; 14 Department of Epidemiology, Radiation Effects Research Foundation, Fukushima, Japan

**Keywords:** Earthquake, Disaster, Nuclear powerplant, Evacuation, Hypertension, Chronic kidney disease

## Abstract

**Background:**

About 146,000 people were forced into long-term evacuation due to the nuclear power plant accident caused by the Great East Japan Earthquake in 2011. Disaster is known to induce hypertension in survivors for a certain period, but it is unclear whether prolonged disaster stress influences chronic kidney disease (CKD). We conducted an observational cohort study to elucidate the effects of evacuation stress on CKD incidence.

**Methods:**

Participants were individuals living in communities near the Fukushima nuclear power plant, aged 40–74 years without CKD as of their 2011 general health checkup (non-evacuees: *n* = 9780, evacuees: *n* = 4712). We followed new-onset CKD [estimated glomerular filtration rate (eGFR) <60 ml/min/1.73 m^2^ or proteinuria] using general annual health checkup data from 2012 to 2014. Association between evacuation and CKD incidence was analyzed using the Cox proportional hazard model.

**Results:**

Mean age of the participants at baseline was 65 years, 46.7% were men, and baseline eGFR was 75.7 ml/min/1.73 m^2^. During the mean follow-up period of 2.46 years, CKD incidence rate was 80.8/1000 and 100.2/1000 person-years in non-evacuees and evacuees, respectively. Evacuation was a significant risk factor of CKD incidence after adjusting for age, gender, obesity, hypertension, diabetes, dyslipidemia, smoking, and baseline eGFR [hazard ratio (HR): 1.45; 95% confidence interval (CI) 1.35–1.56]. Evacuation was significantly associated with the incidence of eGFR <60 ml/min/1.73 m^2^ (HR: 1.48; 95% CI 1.37–1.60), but not with the incidence of proteinuria (HR: 1.21; 95% CI 0.93–1.56).

**Conclusion:**

Evacuation was a risk factor associated with CKD incidence after the disaster.

## Introduction

The Great East Japan Earthquake (9.0 on the Richter scale) and subsequent tsunami took the lives and destroyed the homes of many people on March 11, 2011. The Fukushima nuclear power plant was also damaged, and radiation was leaked into areas along the east coast of Fukushima Prefecture. Survivors living in the area were faced with overwhelming stress from having experienced a large earthquake, massive tsunami, and radioactive leakage all in the short span of a few weeks. Moreover, radioactive contamination is a long-lasting public health concern. The Japanese government-designated areas within a 30-km radius of the plant, as well as areas with high radioactive contamination, as an evacuation zone. People living in the evacuation zone (approximately 146,000 evacuee) were forced to move to another area until further notice.

Disaster has been reported to cause stress-related diseases, particularly hypertension. The elderly and patients with chronic kidney disease (CKD), obesity, and metabolic syndrome are more susceptible to disaster stress [1–3]. Disaster hypertension is characterized as temporal increased blood pressure which returns to pre-disaster levels within 6 months when the disrupted behavioral and biological circadian rhythm is restored. Disaster hypertension is regarded as important because of its association with cardiovascular disease (CVD) and death in the first few weeks or months after the disaster [4–6]. However, it is unknown whether disaster stress can induce chronic diseases such as CKD.

The Fukushima Prefectural Government launched the Fukushima Health Management Survey (FHMS) to investigate the effects of long-term, low-dose radiation exposure among survivors of the Great East Japan Earthquake. Comprehensive health checks are included in the survey with the aim of preventing the development of lifestyle-related diseases such as diabetes, hypertension, dyslipidemia, obesity, and CVD, after the disaster. The FHMS previously reported that prolonged stress changed the lifestyles of the evacuees, resulting in significant increases in the incidence of obesity, hypertension, and diabetes [7–9]. Hypertension and metabolic disorders are well-known predictors of the onset and exacerbation of CKD. Furthermore, the combination of CKD and metabolic syndrome is a strong predictor of the incidence of CVD [10]. Thus, we hypothesized that a natural disaster and subsequent prolonged evacuation stress increases the incidence of CKD and CVD. Here, we investigated the incidence of CKD, and verified the association between evacuation and the incidence of CKD in a longitudinal analysis using annual health checkup data from survivors of the Great East Japan Earthquake.

## Method

### Study population

**Fig. 1:**
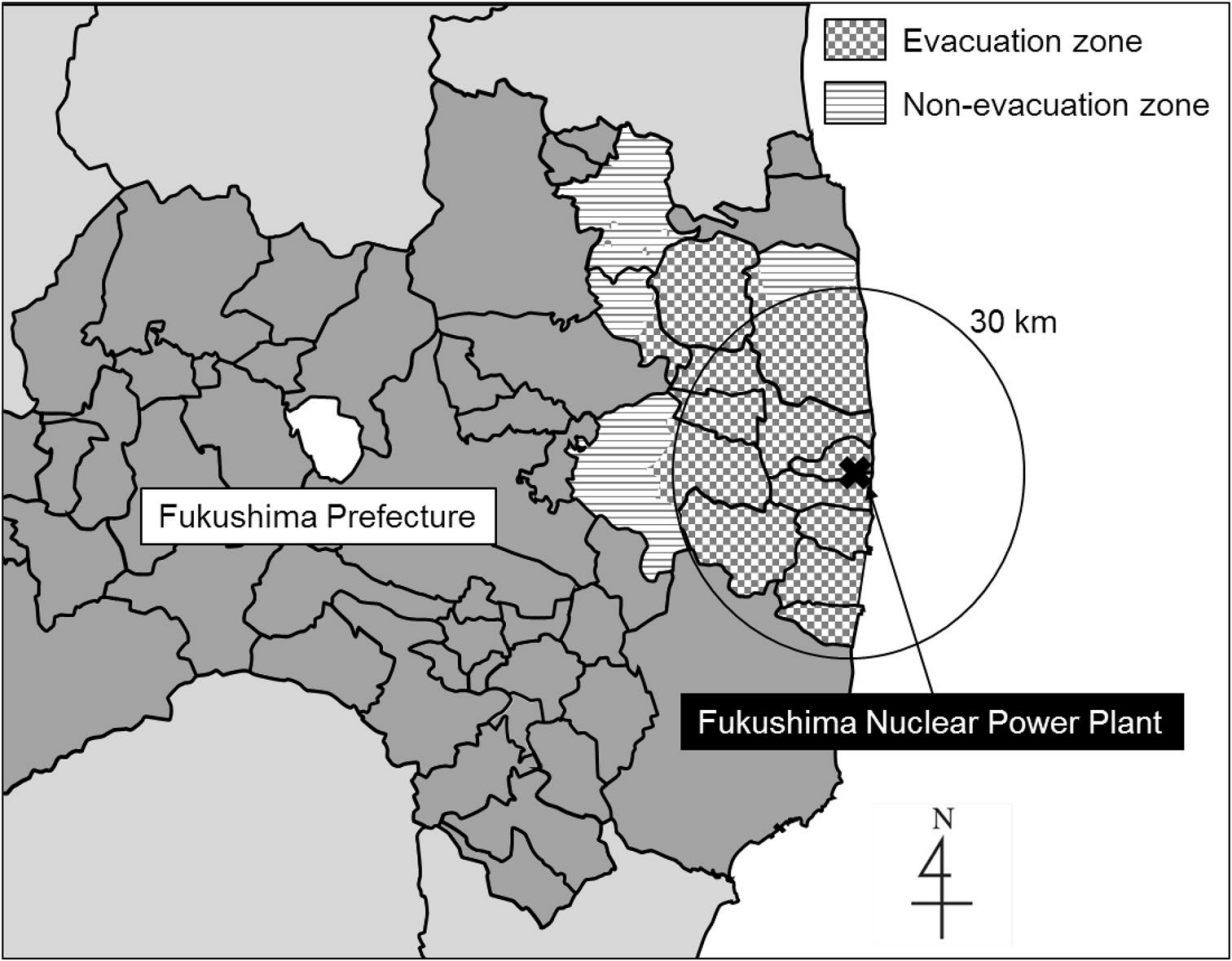
Geographic details of evacuation zone, non-evacuation zone, and the Fukushima Nuclear Power Plant

Participants were Japanese people living one of the following 13 municipalities in Fukushima Prefecture at the time of the disaster: Tamura City, Minami-soma City, Kawamata-machi, Hirono-machi, Naraha-machi, Tomioka-machi, Kawauchi-mura, Okuma-machi, Futaba-machi, Namie-machi, Katsurao-mura, Iitate-mura, and Date City. The government-designated evacuation zone included the entirety of nine of these municipalities (Hirono-machi, Naraha-machi, Tomioka-machi, Kawauchi-mura, Okuma-machi, Futaba-machi, Namie-machi, Katsurao-mura, and Iitate-mura) and some parts of the remaining four. All residents of the evacuation zone were forced to leave their homes after the disaster. Areas not included in the government-designated evacuation zone were defined as the non-evacuation zone in the present study (Fig. 1). The participants were divided into evacuee or non-evacuee based only on the residential area, evacuation zone or not. Evacuation style of evacuee (temporary house or relative house, etc.) and voluntary evacuation from non-evacuation zone were not taken into account.

Since 2008, the Japanese government has conducted an annual health check program, “The Specific Health Check and Guidance System (Tokutei-Kensin)”, which targets those with national health care insurance aged 40–74 years, with the aim of detecting metabolic syndrome. We used data from these annual health checkups from 2011 obtained as part of the comprehensive health checks in the FHMS. A previous report details the methods of the comprehensive health checks and the FHMS [11].

**Fig. 2:**
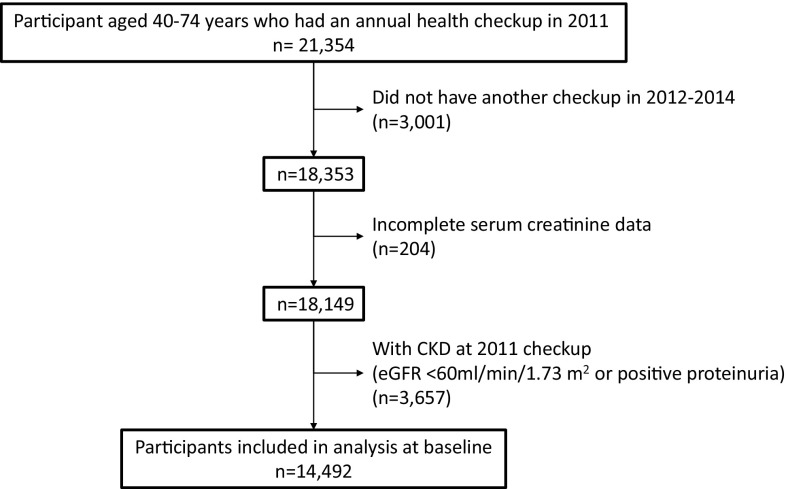
Flow diagram of participants. CKD: chronic kidney disease; eGFR: estimated glomerular filtration rate

Based on national census data, the total population of the 13 municipalities aged between 40 and 74 years was estimated at 125,987 in 2010, the year before the disaster. For the present longitudinal analysis, we targeted residents who received a checkup in 2011 (baseline) and at least one checkup in between 2012 and 2014 (follow-up). Then, we obtained data for the 18,353 residents, representing about 15% of the population from the census data. We also excluded participants with eGFR <60 ml/min/1.73 m^2^ or positive proteinuria by dipstick at baseline. After these exclusions, 14,492 participants were included in the final analyses (Fig. 2).

### Baseline measurements and definitions

Height in stocking feet (m) and weight (kg) in light clothing were measured, and body mass index (BMI) was calculated (kg/m^2^). Underweight, normal weight, and obesity were defined as BMI <18.5, ≥18.5 to <25, and ≥25, respectively. Blood and urine sampling were performed at each local medical institution. Blood pressure (BP) was measured with a standard sphygmomanometer or an automated device by medical staff at each local medical institution. Hypertension was defined as systolic BP ≥140 mmHg, diastolic BP ≥90 mmHg, or the self-reported use of antihypertensive agents. Diabetes was defined as fasting glucose ≥126 mg/dl (7.0 mmol/l), casual glucose ≥200 mg/dl, hemoglobin A1c (HbA1c) ≥6.5%, or the self-reported use of antihyperglycemic agents. We defined dyslipidemia as triglycerides (TG) ≥150 mg/dl and high-density lipoprotein cholesterol (HDL-C) <40 mg/dl, which are widely used values in metabolic syndrome criteria.

Urinalysis by the dipstick method was performed on a single spot urine specimen. Results of proteinuria were recorded as (−), (±), (1+), (2+), and (3+) based on guidelines approved by the Japanese Committee for Clinical Laboratory Standards (http://jccls.org/). In Japan, all urine dipstick tests should be manufactured, so that a urine dipstick result of (1+) corresponds to a urinary protein level of 30 mg/dl. We defined proteinuria as dipstick result of ≥(1+). Serum creatinine was assayed using the enzymatic method. eGFR was estimated using the following equation recommended by the Japanese Society of Nephrology [12]:

eGFR = 194 × serum creatinine^−1.094^ × age^− 0.287^ (×0.739 if female).

In the present study, CKD was defined as eGFR <60 ml/min/1.73 m^2^ or a dipstick proteinuria result of ≥(1+), and new-onset CKD was defined as an endpoint for follow-up.

### Statistical analysis

The participants were divided into two groups based on whether they lived in the evacuation or non-evacuation zone at the time of the disaster: evacuees (*n* = 4712) and non-evacuees (*n* = 9780), respectively. Baseline characteristics of interest (sex, eGFR >75 ml/min/1.73 m^2^, obesity, hypertension, diabetes, dyslipidemia, and smoking habit) were compared between groups using the *χ* ^2^ test. Age, eGFR, BP, and levels of HbA1c, TG, and HDL-C were compared using a non-paired t test. Fasting and causal glucose levels were compared using the Wilcoxon rank sum test. To evaluate the impact of evacuation on the incidence of CKD, the hazard ratio (HR) of new-onset CKD and 95% confidence intervals (CI) for evacuation were calculated using the Cox proportional hazards model with adjustment for other potential confounding factors (age, sex, baseline eGFR, obesity, hypertension, diabetes, dyslipidemia, and smoking). We also assessed the HRs of the incidence of eGFR <60 ml/min/1.73 m^2^ and proteinuria separately. SAS version 9.3 (SAS Institute, Cary, North Carolina, USA) was used for analyses. All probability values for statistical tests were two-tailed, and *p* values <0.05 were considered statistically significant.

## Results

### Baseline characteristics

Among all 18,149 participants at baseline, including those with CKD (*n* = 3657), the prevalence of CKD at baseline was estimated to be 20.2% from Fig. 2. Table 1 shows the baseline clinical characteristics of all participants by group (evacuees or non-evacuees). The mean age of all participants was 65.0 years, women accounted for 53%, and mean eGFR was 75.7 ml/min/1.73 m^2^. Hypertension was present in 54%, diabetes in 10%, and obesity in 30%. First, we compared risk factors of CKD between the two groups at baseline. In the evacuee group, a significantly younger age, higher prevalence of women, and higher levels of baseline eGFR were observed than in the non-evacuee group. However, the evacuee group had a significantly higher prevalence of obesity, diabetes, dyslipidemia, and smoking than the non-evacuee group. No significant difference was observed in the prevalence of hypertension between the two groups.

**Table 1:** Baseline clinical characteristics

	Total	Evacuee	Non-evacuee	*p* value
Number	14,492	4712	9780	
Age (years)	65.0 (9.4)	63.8 (10.6)	65.6 (8.8)	<0.001
Women (%)	53.31	56.07	51.97	<0.001
eGFR (ml/min/1.73 m^2^)	75.7 (10.7)	76.3 (11.1)	75.4 (10.5)	<0.001
≥75(%)	46.0	47.6	45.2	0.005
≥60 to <75(%)	54.0	52.4	54.8	
Obesity^a^(%)	30.22	36.44	27.23	<0.001
BMI(kg/m^2^)	23.5 (3.3)	24.0 (3.5)	23.3 (3.2)	<0.001
Waist circumference(cm)	70.2 (32.1)	71.0 (32.5)	69.8 (31.9)	0.0357
Hypertension^b^(%)	54.02	53.27	54.38	0.21
Blood Pressure (mmHg)				
Systolic	131.7 (16.1)	131.3 (15.9)	131.9 (16.2)	0.0424
Diastolic	77.9 (10.0)	77.7 (9.9)	77.9 (10.1)	0.2932
Diabetes^c^(%)	10.07	11.35	9.46	<0.001
Fasting glucose(mg/dl)	96 (90–104)	97 (91–106)	96 (90–104)	<0.001
Casual glucose (mg/dl)	98 (91–112)	99 (91–111)	98 (91–112)	0.1421
Hemoglobin A1c(%)	5.5 (0.7)	5.5 (0.7)	5.4 (0.6)	<0.001
TG ≥150 or HDL-C <40(%)	22.15	24.89	20.83	<0.001
Triglycerides(mg/dl)	113.8 (73.1)	118.0 (73.2)	111.7 (73.0)	<0.001
HDL-C(mg/dl)	59.8 (14.6)	59.0 (14.5)	60.1 (14.7)	<0.001
Smoking habit(%)	13.32	14.75	12.63	<0.001

Data are expressed means with (SD) or median with (IQR). *p* values were obtained by Chi-square test, Student’s *t* test, and Wilcoxon rank sum test between evacuees and non-evacuees

*SD* standard deviation; *IQR* interquartile range; *BMI* body mass index; *TG* triglycerides; *HDL-C* high-density lipoprotein cholesterol

^a^BMI ≧ 25

^b^Systolic blood pressure ≥140 mmHg, diastolic blood pressure ≥90 mmHg, or self-reported use of antihypertensive agents

^c^Fasting glucose 126 mg/dl, casual blood glucose ≥200 mg/dl, HbA1c ≥6.5%, self-reported use of antihyperglycemic agents

### Incidence of CKD

Next, we investigated the incidence of CKD and the results are shown in Table 2. The cumulative incidence of CKD was 3,093 (21%) over a mean follow-up period of 2.46 years. The incidence rate of CKD of evacuees was higher (100/1000-person-years) than that of non-evacuees by a ratio of 1.24. Since significant differences were observed in baseline characteristics between the two groups, we performed multivariate Cox proportional hazards models. HRs of the risk factors of incidence of CKD are shown in Table 3. Evacuation was a significant risk factor of CKD with a crude HR of 1.38 (95% CI 1.28–1.48) compared with non-evacuation. The multivariate adjusted HR for evacuation was 1.45 (95% CI 1.35–1.56), and it was the highest HR among the other common risk factors of obesity, hypertension, diabetes, and dyslipidemia at baseline. Sex and smoking were not associated with the incidence of CKD.

**Table 2:** Incidence rate of CKD, eGFR <60 ml/min/1.73 m^2^, and proteinuria

	CKD^a^		eGFR <60 ml/min/1.73 m^2^		Proteinuria^b^	
	Incidence	1000 person-years	Incidence	1000 person-years	Incidence	1000 person-years
Total	3093	86.74	2915	81.36	269	6.98
Evacuee	1090	100.18	1036	94.73	92	7.76
Non-evacuee	2003	80.84	1879	75.49	177	6.63

*CKD* chronic kidney desease, *eGFR* estimated glomerular filtration rate

^a^CKD was defined as eGFR <60 ml/min/1.73 m^2^ and/or urinary protein of ≥(1+)

^b^Proteinuria was defined as urinary protein of ≥(1+)

**Table 3:** HRs (95% CIs) for risk of CKD

	Crude HR	Age- and sex-adjusted HR	Multivariable-adjusted HR
Evacuee (ref : non-evacuaee)	1.38 (1.28–1.48)	1.44 (1.33–1.55)	1.45 (1.35–1.56)
Age (continuous)	1.05 (1.05–1.06)	1.05 (1.05–1.06)	1.03 (1.03–1.04)
Women (ref : men)	1.09 (1.01–1.17)	1.20 (1.11–1.28)	1.05 (0.98–1.14)
eGFR			
≥75	Ref	Ref	Ref
≥60 to <75	12.26 (10.74–13.99)	11.08 (9.70-12.65)	11.09 (9.71–12.67)
BMI^a^			
Underweight	0.89 (0.74–1.07)	0.86 (0.72–1.04)	1.08 (0.89–1.30)
Normal weight	Ref	Ref	Ref
Obese	1.33 (1.23–1.43)	1.36 (1.26–1.46)	1.17 (1.09–1.27)
Hypertension (ref : without HT)	1.60 (1.49–1.73)	1.31 (1.22–1.42)	1.26 (1.16–1.36)
Diabetes (ref : without DM)	1.25 (1.12–1.39)	1.21 (1.08–1.34)	1.17 (1.04–1.30)
Dyslipidemia (ref : without DL)	1.20 (1.10–1.30)	1.28 (1.18–1.39)	1.12 (1.03–1.22)
Smoking (ref : no smoking)	0.70 (0.62–0.79)	0.92 (0.81–1.04)	0.99 (0.88–1.13)

*HR* hazard ratio, *CI* confidence interval, *eGFR* estimated glomerular filtration rate, *BMI* body mass index, *ref* reference, *HT* hypertension, *DM* diabetes mellitus, *DL* dyslipidemia

^a^Underweight, normal weight, and obese were defined as BMI <18.5, ≥18.5 to <25, and ≥25, respectively

### Incidence of eGFR <60 ml/min/1.73 m^2^ and proteinuria

We assessed the endpoints of eGFR <60 ml/min/1.73 m^2^ and proteinuria separately, and the results are shown in Table 2. Most of CKD incidence was eGFR <60 ml/min/1.73 m^2^ (*n* = 2,915, mean follow-up period of 2.5 years). It was marked higher than that of positive proteinuria (*n* = 269, mean follow-up period of 2.7 years). As shown in Table 4, evacuation was a significant risk for developing eGFR <60 ml/min/1.73 m^2^ with a multivariate adjusted HR of 1.48 (95% CI 1.37–1.60). Obesity, hypertension, and dyslipidemia at baseline were also significant risk factors. On the other hand, evacuation was not associated with the incidence of proteinuria with a multivariate adjusted HR of 1.21 (95% CI 0.93–1.56). Women had a significantly lower risk of developing positive proteinuria with an HR of 0.58 (95% CI 0.44–0.75). Obesity, hypertension, diabetes, dyslipidemia, and smoking at baseline were also significant risk factors.

**Table 4:** HRs (95% CIs) for risk of eGFR <60 ml/min/ 1.73 m^2^ and proteinuria

	eGFR <60 ml/min/1.73 m^2^		Proteinuria	
	Age- and sex-adjusted HR	Multivariable-adjusted HR	Age- and sex-adjusted HR	Multivariable-adjusted HR
Evacuee (ref : non-evacuee)	1.45 (1.35–1.57)	1.48 (1.37–1.60)	1.35 (1.05–1.74)	1.21 (0.93–1.56)
Age (continuous)	1.06 (1.05–1.06)	1.04 (1.03–1.04)	1.03 (1.02–1.05)	1.03 (1.02–1.05)
Women (ref: men)	1.28 (1.19–1.38)	1.09 (1.01–1.18)	0.45 (0.35–0.58)	0.58 (0.44–0.75)
eGFR				
≥75	Ref	Ref	Ref	Ref
≥60 to <75	19.78 (16.58–23.59)	19.81 (16.60–23.63)	1.02 (0.80–1.31)	1.00 (0.78–1.29)
BMI^a^				
Underweight	0.84 (0.69–1.014)	1.06 (0.87–1.28)	1.10 (0.58–2.09)	1.28 (0.67–2.45)
Normal weight	Ref	Ref	Ref	Ref
Obese	1.31 (1.21–1.42)	1.14 (1.06–1.24)	1.92 (1.51–2.45)	1.55 (1.20–2.00)
Hypertension (ref : without HT)	1.27 (1.17–1.37)	1.22 (1.13–1.33)	2.13 (1.61–2.82)	1.86 (1.40–2.48)
Diabetes (ref : without DM)	1.13 (1.00–1.26)	1.11 (0.99–1.25)	2.49 (1.88–3.31)	2.09 (1.57–2.78)
Dyslipidemia (ref : without DL)	1.25 (1.14–1.36)	1.09 (1.00–1.19)	1.66 (1.28–2.14)	1.38 (1.06–1.79)
Smoking (ref: no smoking)	0.84 (0.73–0.96)	0.91 (0.79–1.04)	1.93 (1.42–2.62)	1.95 (1.43–2.65)

*HR* hazard ratio, *CI* confidence interval, *eGFR* estimated glomerular filtration rate, *BMI* body mass index, *ref* reference, *HT* hypertension, *DM* diabetes mellitus, *DL* dyslipidemia

^a^Underweight, normal weight and obese were defined as BMI <18.5, ≥18.5 to <25, ≥25, respectively

## Discussion

To the best of our knowledge, this is the first longitudinal study of the incidence of CKD among survivors of the Fukushima nuclear power plant accident, and we revealed the impact of evacuation stress on the incidence of CKD after the nuclear power plant accident from 2011 to 2014. This study had two major findings. First, the incidence of CKD after the disaster was high. Second, evacuation was an independent risk factor of the incidence of CKD and other common risk factors.

A Japanese nationwide cohort study was previously conducted to survey the incidence of CKD using data from Tokutei-Kensin. Yano et al. reported that 6.3% of 48,587 participants aged 40–74 years (mean age: 61.7 years; men: 39%) without diabetes developed CKD (eGFR <60 ml/min/1.73 m^2^: 4.1%; proteinuria: 2.5%) over the 3 years from 2008 to 2011 [13]. New onset of eGFR <60 ml/min/1.73 m^2^ in the present study, not of proteinuria, is much higher than above-mentioned study. We did not exclude participants with hypertension, diabetes, and pre-existing CVD, and our participants were older and had a higher proportion of men and obesity compared to the previous study. CKD prevalence at the start of our survey was higher than the previous Japanese nationwide cohort with Tokutei-Kensin data of 2008 (20.2 vs 14.5%: *p* < 0.001) [14]. It is possible that the high-risk target population in this study caused the high incidence. Another cause to consider is the influence of disaster. Not a few peoples with hypertension or diabetes have to interrupt their medication or clinic visits to adjust their medication for a certain period after a disaster, and this could induce temporary change in eGFR. Temporary decreases in eGFR may be counted as new-onset CKD. Furthermore, past natural disasters such as earthquakes and hurricanes have been shown to deteriorate glycemic and blood pressure control even if not accompanied by nuclear power plant accident [1, 15–17]. The FHMS have reported significant increase in prevalence of hypertension, diabetes, and obesity after disaster in both evacuee and non-evacuee [7–9]. Hypertension, diabetes and obesity are well-known risk factors of CKD [18], thus, the high-risk population at baseline and disaster-induced disorders may have synergistically increased the incidence of CKD in both evacuee and non-evacuee.

Evacuation stress cannot be an ignored factor when discussing the influence of a disaster. The FHMS has reported that the prevalence of CKD was not significantly different between evacuees and non-evacuees in 2011 just after the disaster in a cross-sectional study (22.0 vs 21.4%: *p* = 0.37) [19], and in this longitudinal study, we demonstrated that evacuation stress was significantly associated with the incidence rate of CKD with a 1.45-fold higher HR independent of other baseline characteristics. However, it is unclear that how evacuation is associated with CKD based on our study alone. The FHMS for mental health care revealed evacuees who believed that radiation exposure causes negative health effects which were significantly more likely to be psychologically distressed [20]. Moreover, psychological distress was significantly more prevalent among residents of the evacuation zone, even after adjusting for other significant risk factors such as age, gender, living arrangement (own home or other type), experiencing the nuclear power plant accident, loss of a family member, becoming unemployed, and history of mental illness. Interestingly, psychological distress in each evacuation zone was positively associated with the radiation levels in the evacuees’ environment [21]. These studies suggest that fear of radiation risk contributes to psychological distress among evacuees. Although not under natural disaster conditions, a prospective population-based study in Finland (*n* = 466) showed that high psychological distress at baseline predicts the development of metabolic syndrome independent of age, gender, marital status, educational attainment, and baseline health behaviors (smoking, alcohol use and leisure time physical activity) with an odds ratio of 1.83 (95% CI 1.05–3.21) [22]. Evacuation has been demonstrated to be significantly associated with increased incidence of metabolic syndrome by the FHMS [23]. Moreover, FHMS have revealed that evacuation was a significant risk for overweight and diabetes with an HR of 1.61 (95% CI 1.47–1.77) and 1.39 (95% CI 1.20–1.63), respectively [7, 8]. FHMS has speculated that these increasing metabolic disorders were induced by a less physical activity due to loss of jobs and social connectedness by evacuation.

Evacuation also has been reported to be significant risk of hypertension especially among men with HR 1.24 (95% CI 1.1–1.39) by FHMS [9]. Therefore, we could regard evacuation as a composite risk factor mainly composed of metabolic disorders and hypertension. Kario stated that people with psychological distress or metabolic syndrome are more susceptible to disaster hypertension. Disaster hypertension was accompanied with increased salt sensitivity, blood viscosity, platelet activation, and endothelial dysfunction caused by sympathetic nerve activation [2]. These conditions might be involved in the CKD incidence that was based on eGFR <60 ml/min/1.73 m^2^, not proteinuria. Taking the results of the previous studies together with our results, evacuation could induce prolonged psychological distress and less physical activity which increase new-onset or exacerbation of metabolic disorders and hypertension, and it may cause the high incidence of CKD. Japanese government has begun to release gradually the evacuation zone with decontamination. However, there are unanswered questions as to how long it takes for the influence of the disaster to weaken and further observation on this topic is needed.

The strengths of the present population-based study are its relatively large sample size and the incorporation of complete eGFR and proteinuria information at baseline and at each annual checkup. However, there are also several limitations. First, we did not follow residents who received no annual checkup or only one annual checkup. This selection bias may have affected our results. Second, our study did not include data collected before the disaster as a control. Serum creatinine had not been examined at annual checkups in most areas before 2011. Cohorts in areas not affected or less affected by the disaster who underwent checkups should be investigated to evaluate the influence of the disaster. Third, we could not exclude one participant who was temporarily positive for CKD, and this may have led to overestimation of the incidence of CKD as long as using annual checkup data. Several checkups in a year are necessary to determine a more accurate incidence of CKD; however, it is difficult for general population to receive frequent checkups in a year. Finally, we used baseline clinical characteristics and evacuation as confounders in our multivariate analysis. We cannot completely rule out the possibility of residual confounding, for example, socioeconomic factors such as changes in living conditions and job status after the disaster. The target population consisted of people with national health insurance (mainly intended for farmers, fisherpersons, the self-employed, and retirees), and we did not include people covered by social insurance (mainly intended for employees) in our analyses. These socioeconomic factors may have influenced the association between evacuation and risk of CKD.

## Conclusion

In conclusion, evacuation was a risk factor strongly associated with the incidence of eGFR <60 ml/min/1.73 m^2^ among residents around the damaged Fukushima nuclear power plant over 3 years. In addition to environmental recovery, care for psychological distress, physical activity, and following clinical disorders might be necessary to prevent CK among evacuees.
